# Ustekinumab or Vedolizumab after Failure of Anti-TNF Agents in Crohn’s Disease: A Review of Comparative Effectiveness Studies

**DOI:** 10.3390/jcm13082187

**Published:** 2024-04-10

**Authors:** Mohmmed Tauseef Sharip, Nilanga Nishad, Lushen Pillay, Nilkantsingh Goordyal, Samuel Goerge, Sreedhar Subramanian

**Affiliations:** Department of Gastroenterology, Cambridge University Hospital NHS Foundation Trust, Cambridge CB2 0QQ, UK; mohammed.sharip@nhs.net (M.T.S.); nilanga.nishad@nhs.net (N.N.); lushen.pillay@nhs.net (L.P.); drngoordyal@gmail.com (N.G.); samogeorge@gmail.com (S.G.)

**Keywords:** inflammatory bowel disease, Crohn’s disease, vedolizumab, anti-tumour necrosis factor antibody, ustekinumab

## Abstract

**Background:** Anti-tumour necrosis factor (TNF) agents are effective in Crohn’s disease (CD), but some patients lose responsiveness and require alternative biologic therapy. Until recently, ustekinumab and vedolizumab were the only other biological agents approved for use in CD. There are no randomised trials which compare the efficacy of these two agents in patients with anti-TNF refractory disease, but several retrospective cohort studies have compared their effectiveness in this setting. **Aim:** To review the effectiveness of ustekinumab and vedolizumab in anti-TNF refractory patients with CD. **Methods:** We included studies that compared the effectiveness of ustekinumab and vedolizumab in treating patients with anti-TNF refractory CD. We recorded the sample size, primary and secondary outcome measures and whether the studies employed adjustments for appropriate confounders. **Results:** Fourteen studies were included with a total sample size of 5651, of whom 2181 (38.6%) were treated with vedolizumab and the rest were treated with ustekinumab (61.4%). Of the fourteen studies included, eight found ustekinumab to be more effective in achieving clinical remission/steroid-free remission in the induction phase or during maintenance therapy (at least 1-year post-treatment) or that treatment persistence rates with ustekinumab were higher than with vedolizumab. Only one study reported vedolizumab to be superior during the maintenance phase in terms of clinical remission or treatment persistence rates. Biochemical outcomes were reported in five studies, two of which showed superiority for ustekinumab at 14 weeks and the other at 52 weeks. Only two studies reported endoscopic and/or radiologic outcomes; of these, one study showed ustekinumab to be significantly better at achieving endoscopic and radiologic responses. Adverse outcomes were broadly comparable, barring a single study which reported a lower hospitalisation rate for severe infection with ustekinumab. **Conclusions:** Most studies found ustekinumab to be more effective or non-inferior to vedolizumab in treating patients with anti-TNF refractory CD. Although many studies adjusted appropriately for confounders, the possibility of residual confounding remains and further data from prospective studies are warranted to confirm these findings. Further studies are required to compare these two therapies to other emerging therapies, such as Janus-kinase inhibitors.

## 1. Introduction 

Over the past two decades, there has been a significant expansion in the therapeutic armamentarium of Crohn’s disease (CD). This revolution was led initially by the anti-tumour necrosis factor (TNF) antibody infliximab, which was approved in 1998, followed by adalimumab in 2007. Anti-TNF agents were the mainstay of advanced therapy for CD for several years. There was no other class of therapies approved for almost two decades until the introduction of vedolizumab in 2014 and subsequently ustekinumab in 2016.

Vedolizumab is a monoclonal antibody specifically targeting the α_4_β_7_ integrin; it blocks the interaction between α_4_β_7_ integrin and MAdCAM-1, selectively inhibiting gastrointestinal inflammation [1]. Ustekinumab binds to the p40 subunit common to IL-12 and IL-23 and prevents their interaction with the IL-12 receptor β1 subunit of the IL-12 and IL-23 receptor complexes which subsequently modulate lymphocyte function [2].

More recently, patients and clinicians have other options, such as the selective P19 antibody risankizumab and the selective Janus kinase-1 (JAK-1) inhibitor upadacitinib. However, risankizumab and upadacitinib were only approved in 2022 and 2023, respectively, and thus, until recently, patients who failed anti-TNF therapies were treated with either ustekinumab or vedolizumab. 

Anti-TNF agents have transformed the treatment of CD and are typically used as first-line advanced therapies in CD unless there are contraindications. The availability of low-cost infliximab and adalimumab biosimilars has further cemented their position as first-line advanced therapies for CD in several healthcare settings. Around 10–30% of patients, however, fail to respond to initial therapy, and up to 40% subsequently lose responsiveness or develop limiting side-effects requiring alternative biological therapy [3]. Until recently, the α4β7 antibody vedolizumab, and the p-40 antibody ustekinumab, were widely used after the failure of anti-TNF therapies. In a randomised trial of anti-TNF refractory CD, vedolizumab demonstrated efficacy for the induction and maintenance of remission [4]. Similarly, ustekinumab was superior to the placebo in achieving clinical response and remission in randomised trials of patients with anti-TNF refractory CD [5]. Data from clinical trials appear to suggest broadly comparable efficacy and safety for both agents in treating patients with anti-TNF refractory CD. For instance, the induction response rates (defined by a reduction of 100 points in the CD activity index) was 39.2% (placebo 22.3%) for vedolizumab [4] and 37.8% (placebo 20.2%) for ustekinumab [5], respectively.

Similarly, the overall severe adverse event rate was 6% (placebo 8%) for vedolizumab [4] and 7.2% (placebo 6.1%) for ustekinumab [5]. No randomised controlled trials have been performed to compare the two agents’ efficacy in this setting. Several retrospective real-world studies have compared the effectiveness of these two agents in this setting with contradictory findings [6,7]. In light of this, we sought to review the effectiveness of vedolizumab and ustekinumab in treating patients with anti-TNF refractory CD.

## 2. Methods

We conducted a PubMed, Google Scholar, EMBASE, and Cochrane library search for all papers in which patients received vedolizumab or ustekinumab after anti-TNF failure for CD. For the PubMed search, we used the following search criteria: vedolizumab AND ustekinumab OR (Interleukin-12) OR (Interleukin-23) AND (Crohn’s Disease) OR (Inflammatory Bowel Disease). Our inclusion criteria were 1. CD studies written in the English language. 2. Studies that reported an outcome (clinical and or biochemical/endoscopic response) following treatment with vedolizumab and ustekinumab in patients who failed anti-TNF therapy. We excluded any studies in which 1. there was no prior anti-TNF exposure; 2. there was no literature review; 3. patients had already received either vedolizumab or ustekinumab as their first-line therapy; 4. there was any network meta-analysis. We extracted data on both primary and secondary outcome measures as reported by the studies. Efficacy was typically based on clinical parameters across most studies. Some studies reported data on endoscopic and biochemical improvement (C-reactive protein and faecal calprotectin measurements) as secondary outcome measures. Finally, we collected data on adverse events. Due to variations among studies on time points of assessment of response, we categorised the induction phase as <4 months and maintenance phase as >4 months. Given the heterogeneity across studies, we performed a narrative instead of a systematic review. 

## 3. Results

### 3.1. Characteristics of Included Studies

We included 14 studies with a total sample size of 5651, of which 2181 (38.6%) were treated with vedolizumab and 3470 (61.4%) with ustekinumab. Most studies had a follow-up period of 1 year (48–54 weeks), and three studies had a follow-up of more than 2 years.

All the studies looked at patients’ baseline demographic, including age; sex; smoking status; the median age of diagnosis and starting using both vedolizumab and ustekinumab; disease location; disease phenotype; concomitant medication history, particularly of steroids and immunomodulators; number and line of anti-TNF medication exposure; disease activity using a validated scoring system like CDAI; presence of concomitant perianal disease; and previous surgical history. Some studies additionally looked at biomarkers like CRP, haemoglobin, and faecal calprotectin. A limited number of studies looked at their baseline endoscopic severity score. Baseline demographics were broadly similar across the studies. However, some inter-study variation was observed. Four studies reported a baseline BMI [8,9,10,11], and one study reported body weight [12] which was equally distributed across both groups. Ten included studies corrected for imbalances among baseline variables between ustekinumab- and vedolizumab-treated patients using propensity score matching [7,8,12,13,14,15,16,17,18,19]. Table 1 provides a summary of the included studies. 

### 3.2. Induction Phase—Clinical Response, Remission, and Steroid-Free Clinical Remission 

Among the studies that reported clinical outcomes at weeks 14–20 (induction period) (Table 2A), four studies showed superior clinical response, treatment persistence, and/or clinical steroid-free remission to be higher in patients treated with ustekinumab [8,14,18,20]. The sample size for these studies ranged from 130 to 835, and three of these studies adjusted for confounders using propensity weighting [9,11,19]. Townsend et al.’s study showed that the steroid-free remission rate was higher among patients treated with ustekinumab after propensity matching [9]. Garcia et al. also showed higher clinical remission and steroid-free clinical remission in patients treated with ustekinumab after the induction period [19]. In the other two studies, though, patients treated with ustekinumab had numerically higher clinical or steroid-free remission rates that were not statistically significant after propensity adjustment [14,20]. Among the studies that reported the biochemical outcomes during induction (Table 3), only one reported a higher deep clinical remission (steroid-free clinical remission and faecal calprotectin < 100 µg/g) rate in patients treated with ustekinumab [14]. 

### 3.3. Maintenance Phase—Clinical Response, Remission, and Steroid-Free Clinical Remission

Eight studies reported ustekinumab was superior to vedolizumab in achieving clinical response and/or steroid-free clinical remission or treatment persistence (Table 2B) [7,8,11,12,14,17,18,20]. Four studies did not find any difference in outcome among both groups [9,10,13,19]. Only one study by an Italian group reported that vedolizumab was better at achieving clinical responses in patients with anti-TNF refractory CD [16]. However, both groups had similar objective response rates and remission (measures using endoscopy/MRI/ CT scan/US small bowel). Studies reported biochemical steroid-free remission showed ustekinumab to be either superior or non-inferior in achieving remission (Table 3). A large study by Yang et al. which included 536 patients, also looked at endoscopic remission at the end of week 52, which showed that ustekinumab was superior to vedolizumab (31.4% vs. 12.7%, *p* < 0.001) [21].

### 3.4. Predictors of Response and Remission 

In general terms, most studies demonstrated, to a varying degree of significance, the following high-risk findings to be associated with a higher risk for not reaching clinical responses: young age at disease onset, longer disease duration, high CRP at baseline, steroids at baseline, complicated phenotype, severe disease with high baseline Harvey–Bradshaw Index (HBI score), exposure to more than one anti-TNF, and smoking. For instance, Hyun et al. reported that a diagnosis of CD after age 40 was significantly predictive of clinical remission, while concomitant steroid use and a longer duration of disease predicted non-clinical remission [10]. Onali et al. found the use of immunomodulators or steroids at baseline, moderate to severe disease activity, and previous surgery related to CD to be associated with non-response for patients treated with ustekinumab, while for vedolizumab baseline disease activity was the only significant negative predictor, and for both drugs, a clinical response at 26 weeks predicted steroid-free remission at 52 weeks [16]. They also found that patients treated with vedolizumab were more likely to achieve steroid-free remission if they were younger than 40, had no proximal disease involvement or steroids at baseline, and had a history of perianal disease [16]. 

### 3.5. Perianal Fistula Healing 

Three studies reported clinical outcomes of perianal disease treated with either ustekinumab or vedolizumab (Table 4) [14,20,22]. Two studies showed no significant difference between patients treated with ustekinumab and vedolizumab [14,20]. A recent large retrospective study reported better clinical responses and remission rates of active perianal fistula in patients treated with ustekinumab compared to vedolizumab or a second anti-TNF agent after the failure of a first anti-TNF agent [23]. In patients with inactive perianal disease, ustekinumab, when used as a second-line agent, showed no significant difference in recurrence rates compared to vedolizumab, although the overall event rates were low.

### 3.6. Adverse Events and Safety 

A total of nine studies reported adverse events (Table 5). The most reported adverse events were infections which ranged from minor to severe. Most of these studies, except one, did not report a significant difference between the total adverse events, infection-related adverse events, and treatment discontinuation due to adverse events during the study period between patients treated with vedolizumab and ustekinumab. A single study reported a significantly lower incidence of hospital admission due to infections (adjusted HR 0.56, 95% CI 0.34–0.92) [17]. Across all studies, for infection-related adverse events, the range was 3.8% to 28.7% for vedolizumab and 2% to 12.5% for patients treated with ustekinumab. Total adverse events ranged from 1.5% to 47.7% for vedolizumab and 0% to 24.5% for ustekinumab. The most frequently recorded parameter was the discontinuation of therapy due to adverse events. The mean rate of discontinuation of therapy due to adverse events was 4.9% for patients treated with vedolizumab and 2.7% for patients treated with ustekinumab.

## 4. Discussion

Several retrospective studies have now compared the effectiveness of ustekinumab and vedolizumab in patients with anti-TNF refractory CD in a real-world setting. The majority of the studies found that ustekinumab was broadly more effective than vedolizumab in terms of clinical remission rates, treatment persistence rates, and biochemical improvement rates. A few studies did not report any significant differences between those two drugs in terms of clinical and biochemical response rates [9,10,15]. Interestingly, a single study showed vedolizumab to be more effective in achieving clinical and steroid-free clinical remission and treatment persistence than ustekinumab [16]. These findings are consistent with a previous meta-analysis, which showed ustekinumab to be superior to vedolizumab in achieving steroid-free clinical remission, biologic remission, and treatment persistence at week 52 [24]. The reasons for the discrepant findings across the studies are most likely explained by differences in the sample size and patient characteristics including the number of lines of anti-TNF exposure and phenotypic differences among the included patients. Although many studies corrected for these imbalances using propensity weighting, it is likely that residual confounding may account for these differences. Most studies did not report a difference in the frequency of adverse events between the two drugs; severe infection rates were similar between the two groups apart from a single study.

Nine studies reported clinical or steroid-free clinical remission after the induction doses of ustekinumab and vedolizumab (Table 2A) [8,11,12,13,14,15,16,18,20]. Four studies reported that ustekinumab was superior in achieving clinical remission after induction therapy compared to vedolizumab [8,14,18,20], and the rest (five studies) did not show any statistical differences in outcome after induction therapy [10,11,12,13,15]. This apparent contradictory result could partially be explained by the frequent use of additional doses of vedolizumab during the induction period [21,24,25]. In addition, there is likely to be further confounding from the concurrent use of steroids and immunomodulators during the induction phase. Importantly, the time point for the assessment of response varied between studies. The measurement of responses at earlier time points may have underestimated the effect of vedolizumab, as a difference in efficacy was only noted at week 10 in the pivotal clinical trial of vedolizumab in patients with anti-TNF refractory CD [25].

Among studies which reported clinical outcomes at the end of the first year (week 48–week 52) of treatment (Table 2B), eight studies showed a superior clinical response rate and or steroid-free clinical remission for ustekinumab compared to vedolizumab [7,8,11,12,14,17,18,20], four studies showed no difference in the clinical response rate or steroid-free clinical remission between both drugs [9,10,13,19], and only a single study showed that vedolizumab achieved a superior clinical response rate [16]. However, in that study by Onali et al., no differences were observed when objective markers of inflammation were considered [16]. A few studies reported factors associated with treatment response. For ustekinumab, efficacy was higher in patients with ileal disease, a penetrating phenotype, and those on combination therapy [12]. Factors associated with vedolizumab failure were age > 35 years, non-complicated phenotype, no prior bowel resection, and no steroids at baseline [14]. This is consistent with similar reports of better efficacy for vedolizumab in patients without prior resection and a non-complicated disease phenotype [26,27]. Notably, many of the studies did not report an association between clinical factors and treatment response, which may be related to sample size differences between the studies.

Although many studies only reported clinical outcomes, a few studies reported biochemical outcomes, and endoscopic outcomes were reported in a single study (Table 3). Among the five studies which reported biochemical parameters (CRP and/or faecal calprotectin) [7,12,13,14,20], two studies reported a difference, both in favour of ustekinumab [13,14]. Only two studies reported mucosal healing as their outcome [7,9], and of these, one study showed a significant difference in favour of ustekinumab for endoscopic response and remission at both weeks 26 and 52 [7]. The same study reported that radiological improvement rates were also better with ustekinumab at weeks 26 and 52. To assess mucosal healing, both studies used simplified endoscopic scoring for CD (SES-CD) and intestinal ultrasound and/or CT and MRI to assess for transmural healing.

A sub-group of particular interest is patients with perianal CD. There are no randomised trials of ustekinumab or vedolizumab in perianal CD, and most of the data is from a post hoc analysis of clinical trials or real-world data. There are scant real-world data on the efficacy of ustekinumab and vedolizumab on perianal CD. In a post hoc analysis of GEMINI-2, the perianal fistula closure rate was higher in the treated group compared to the placebo group [27]. Similarly, a post hoc analysis of other ustekinumab registrational trials reported that 24.7% of patients achieved fistula closure at week 8, and 80% achieved clinical fistula response at week 44 after ustekinumab treatment [28]. A few studies included in our review specifically reported outcomes in perianal CD (Table 4). Manley et al. included 56 patients with perianal disease (39 in the ustekinumab group and 17 in the vedolizumab group) [14]. Overall, there was no statistically significant difference between these two groups. In the Bacsur et al. cohort, 44.72% in the ustekinumab group and 24.62% in the vedolizumab group had perianal disease [20] and similarly did not report a difference between the two agents. A recent multi-centre study reported fistula response and remission rates in patients with anti-TNF failure and perianal CD. Interestingly, significantly better outcomes were reported for ustekinumab compared to both vedolizumab and a second anti-TNF agent [22]. These figures are broadly comparable to previously reported response rates for vedolizumab [27,29] and ustekinumab [28].

Several studies reported safety outcomes, and most of these studies had no significant differences between both groups (Table 5). In the multi-centre study by Lenti, the overall infection rate was numerically higher in patients treated with ustekinumab, but this was not adjusted for frailty or co-morbidities [15]. However, a single study by Kappelman, which compared 1217 and 667 new users of ustekinumab and vedolizumab, respectively, demonstrated a lower rate of infection-related hospitalisation for ustekinumab [17]. The slight variation in infection risk noted among the studies could largely be explained by differences in co-morbidities, frailty, and concomitant steroid therapy between the patient populations. It is now well recognised that co-morbidities [30] and frailty [31] rather than age dictate infection risk with biologic therapy.

It is important to note that the included studies have some broad limitations. The studies are retrospective and non-randomised and do not account for inherent treatment selection bias. Although some studies attempted to adjust for potential confounding between treatment groups by applying inverse probability weighting to provide unbiased treatment effect estimates, the validity of this analysis relies on the untestable assumption that all confounders have been accounted for. Endoscopic follow up data were not included in many studies, and, therefore, there are limited data on mucosal healing. Similarly, the biochemical parameters were also not reported widely. Another limitation is that minor adverse events and infections might not have been reported by patients as the information is captured retrospectively; therefore, the results may underestimate the incidence of adverse events. The duration of follow-up is also limited, and ideally studies with longer follow-up durations are required to confirm outcomes over a prolonged period. Finally, none of the studies presented outcomes following failure of vedolizumab or ustekinumab—specifically, there were no reports of the long-term outcome of patients after failure of second-line therapy. Notwithstanding these limitations, well-conducted retrospective studies with propensity adjustment are increasingly used in areas where there are no randomised controlled trials. However, further randomised trials are required to inform the optimal sequencing of therapies in IBD, and these studies should also incorporate recently approved therapies.

## Figures and Tables

**Table 1:** Characteristics of included studies.

Author	Year	Follow up Duration	Sample Size	Propensity Adjustment	Conclusions
Alric et al. [12]	2020	48 weeks	Vedolizumab: *n* = 132	Yes	Higher rate of clinical remission and treatment persistence for ustekinumab at 12 months
			Ustekinumab: *n* = 107		
Townsend et al. [8]	2020	12 months	Vedolizumab: *n* = 85	Yes	Ustekinumab more effective than vedolizumab at end of induction (2 months) and at 12 months
			Ustekinumab: *n* = 45		
Biemans et al. [13]	2020	52 weeks	Vedolizumab: *n* = 128	Yes	Ustekinumab showed superior effectiveness at 52 weeks
			Ustekinumab: *n* = 85		
Manlay et al. [14]	2021	54 weeks	Vedolizumab: *n* = 88	Yes	Ustekinumab more effective than vedolizumab at week 54
			Ustekinumab: *n* = 224		
Lenti et al. [15]	2021	52 weeks	Vedolizumab: *n* = 118	Yes	Higher clinical remission rates after induction for ustekinumab but no difference at 52 weeks
			Ustekinumab: *n* = 275		
Onali et al. [16]	2022	52 weeks	Vedolizumab: *n* = 231	Yes	Comparable clinical effectiveness after 26 weeks of treatment. Higher rate of clinical remission at 1 year for vedolizumab
			Ustekinumab: *n* = 239		
Bacsur et al. [20]	2022	52 weeks	Vedolizumab: *n* = 65	No	Higher remission and treatment persistence rates for ustekinumab
			Ustekinumab: *n* = 161		
Rayer et al. [11]	2022	118 weeks	Vedolizumab: *n* = 42	No	Short-term efficacy rates similar but long-term treatment persistence lower for ustekinumab
			Ustekinumab: *n* = 90		
Hyun et al. [10]	2022	48 weeks	Vedolizumab *n* = 28 Ustekinumab *n* = 16	No	Vedolizumab and ustekinumab equally effective
Alrashed et al. [9]	2023	52 weeks	Vedolizumab: *n* = 29		Ustekinumab and vedolizumab both equally effective (numerical superiority for ustekinumab)
			Ustekinumab: *n* = 101		
Kappelman et al. [17]	2023	52 weeks	Vedolizumab: *n* = 490	Yes	No difference in treatment persistence at 52 weeks, but lower rates of hospitalisation for CD and infection
			Ustekinumab: *n* = 885		
Garcia et al. [18]	2023	4.7 years	Vedolizumab: *n* = 207	Yes	Remission, steroid-free remission, and durability higher with ustekinumab
			Ustekinumab: *n* = 628		
Kapizioni et al. [19]	2023	3 years	Vedolizumab: *n*= 388	Yes	No significant difference between ustekinumab and vedolizumab
			Ustekinumab: *n* = 228		
Yang et al. [7]	2023	3 years	Vedolizumab: *n* = 150	Yes	Ustekinumab was superior to vedolizumab with superior clinical and objective responses
			Ustekinumab: *n* = 386		
			Total-5651		
			Vedolizumab: *n* = 2181		
			Ustekinumab: *n* = 3470		

**Table 2:** (A): Induction response, remission, steroid-free remission, and treatment persistence rates among patients treated with ustekinumab and vedolizumab. (B): Maintenance of remission, steroid-free remission, and treatment persistence rates among patients treated with ustekinumab and vedolizumab.

(A)						
**Study**	**Clinical Response Measures**	**UST**		**VDZ**		**Sig**
		***n***	**%**	***n***	**%**	
Alric et al. [12]						
UST—(*n* = 107)	Clinical remission	45	42.3	61	46.1	0.59
VDZ—(*n* = 132) W 14	SFCR	41	38.2	45	34.4	0.57
Bacsur et al. [20]	SFCR	156	96.94	30	46.3	0.18
UST—(*n* = 161)						
VDZ—(*n* = 65)	Treatment persistence	139	86.5	38	57.9	<0.001
Induction period 16–20 weeks						
Biemans et.al. [13]	SFCR	17	20.3	37	29	0.327
UST—(*n* = 85)						
VDZ—(*n* = 128)						
W 12						
Garcia et al. [18]	Clinical response	293	46.7	64	30.9	<0.001
UST—(*n* = 628)	Clinical remission	242	38.5	49	23.7	<0.001
VDZ—(*n* = 207) W 16	SFCR	223	35.5	45	21.8	<0.001
Hyun.et al. [10]						
UST—(*n* = 16)	Clinical remission	8	50	15	53.57	0.82
VDZ—(*n* = 28) W 16						
Lenti et al. [15]						
UST—(*n* = 281)	Clinical remission	274	97.51	113	95.76	0.631
VDZ—(*n* = 118) Induction period 3 months						
Manlay et al. [14]						
UST—(*n* = 224)	Deep remission (SFCR + FC < 100)	58	25.9	3	3.8	0.02
VDZ—(*n* = 88) W 14						
Rayer et al. [11]						
UST—(*n* = 90)	Clinical remission	26	29	16	38	0.54
VDZ—(*n* = 42) Induction period 14–24 weeks						
Townsend et al. [8]						
UST—(*n* = 45)	2 months—SFCR	13	28.89	10	11.76	0.015
VDZ—(*n* = 85) Induction period of 2 months followed by 4 months follow up	2 months—clinical remission	16	35.56	14	16.47	0.014
	2 months—clinical response	22	48.89	30	35.29	0.132
	4 months—SFCR	17	37.78	17	20	0.028
	4 months—clinical remission	18	40	18	21.18	0.023
	4 months—clinical response	25	55.56	33	38.82	0.068
(B)						
**Study**	**Clinical Response Measurements**	**UST**		**VDZ**		**Sig**
		***n***	**%**	***n***	**%**	
Alrashed et al. [9]	Hospitalised	26	25.74	7	24.14	NR
UST—(*n* = 101)	IBD-related surgery	12	11.88	11	37.93	NR
VDZ—(*n* = 29) Outcomes at W52	At least one steroid course	16	15.84	14	48.28	NR
Alric et al. [13]	Clinical remission	58	54.4	51	38.3	0.03
UST—(*n* = 107)	SFCR	48	44.7	45	34	0.13
VDZ—(*n* = 132) Outcomes at W48	Treatment persistence	76	71.5	66	49.7	<0.01
	SFCR	29	27.4	23	17.2	0.09
	CD-related surgery	10	9.5	14	10.6	0.81
	Hospitalisation	26	24.1	42	32.1	0.21
	Dose optimisation	32	30.1	71	53.5	<0.01
Bacsur et al. [20]	SFCR	88	54.46	26	39.68	0.008
VDZ—(*n* = 65)						
UST—(*n* = 161)	Treatment persistence	21	13.04	27	41.54	<0.001
Outcomes at W52						
Biemans et al. [13]						
UST—(*n* = 85)	W 24—SFCR	36	42	39	30.4	0.215
VDZ—(n = 128)	W 24—clinical remission	39	46.4	37	29	0.043
Outcomes at W24 and W52	W 52 combined clinical/biochemical remission	23	27.1	14	10.7	0.031
Garcia et al. [18]	W 26—clinical response	380	60.5	95	45.7	<0.05
UST—(*n* = 628)	W 52—clinical response	342	54.5	78	37.6	<0.001
VDZ—(*n* = 207) Outcomes at W 26, W 52, and W 156	W 156—clinical response	152	24.2	29	14.2	<0.05
	W 26—clinical remission	337	53.6	79	38.4	<0.001
	W 52—clinical remission	302	48.1	46	22	<0.001
	W 156—clinical remission	202	32.2	28	13.4	<0.05
	W 26—SFCR	315	50.1	70	33.8	<0.05
	W 52—SFCR	291	46.3	46	22	<0.001
	W 156—SFCR	185	29.5	25	11.9	<0.05
Hyun et al. [10]	Clinical remission	Survival curve analysis				0.692
UST—(*n* = 16)						
VDZ—(*n* = 28) Outcome at W 48						
Kapizioni et al. [19]	Treatment persistence at 1 year	238	56	474	63.8	0.599
VDZ—(*n* = 743)						
	Treatment persistence at 2 years	71	16.71	208	27.99	
UST—(*n* = 425) Outcomes at W 52, W 104 and W 156	Treatment persistence at 3 years	23	5.41	79	10.63	
Lenti et al. [15]						
UST—(*n* = 281)						
VDZ—(*n* = 118)	Clinical remission	215	76.51	78	66.1	0.157
Outcomes at W 52						
Manlay et al. [14]						
UST—(*n* = 224)	SFCR	110	49.3	37	42.2	0.04
VDZ—(*n* = 88) Outcome at W 54						
				
				
Onali et al. [16]	W 26 clinical response	144	60.1	151	65.4	0.277
	W 26 clinical remission	101	42.1	103	44.8	0.596
	W 26 SFCR	92	38.3	94	40.7	0.636
	W 52 clinical response	154	64.6	158	68.4	0.426
UST—(*n* = 239)	W 52 clinical remission	102	42.5	128	55.5	0.01
VDZ—(*n* = 231)	W 52 SFCR	09-Jul	40.6	118	51.1	0.038
Outcomes at W 26 and W 52						
Rayer et al. [11]	W 26—treatment persistence	68	75	36	86	
UST—(*n* = 90)	W 52—treatment persistence	46	51	32	75	
VDZ—(*n* = 42)	W 104—treatment persistence	18	20	24	57	
Outcomes at W 26, W 52, and W 104						
Townsend et al. [8]	6 months—clinical response	22	48.89	33	38.82	0.269
UST—(*n* = 45)	6 months—clinical remission	18	40	14	16.47	0.003
VDZ—(*n* = 85)	6 months—SFCR	17	37.78	13	15.29	0.004
Outcomes at W 26 and W 52						
	12 months—clinical response	24	53.33	37	43.53	0.287
	12 months—clinical remission	19	42.22	22	25.88	0.057
	12 months—SFCR	19	42.22	21	24.71	0.04
Yang et al. [7]	W 26—SFCR		46		33.4	0.003
UST—(*n* = 194)	W 26—clinical remission		47		37.7	0.015
VDZ—(*n* = 84) Outcomes at W 26 and W 52	W 52—clinical remission		47		37.7	0.015
	W 52—SFCR		55.6		29.7	<0.001
Kappelman et al. [17]	Treatment persistence	404		205	RR; 1.09	(0.95–1.25)
UST—(*n* = 884)	All-cause hospitalization	213		156	HR; 0.73	(0.59–0.91)
VDZ—(*n* = 484)	Hospitalization for CD without surgery	66		64	HR; 0.56	(0.4–0.83)
Outcomes at W 52	Hospitalization for CD with surgery	76		47	HR; 0.83	(0.57–1.22)

UST: ustekinumab; VDZ: vedolizumab; SFCR: steroid-free clinical remission.

**Table 3:** Biochemical and endoscopic outcomes at induction and during maintenance.

**Study**	**Clinical Response Measurement**	**UST**		**VDZ**		**Sig**
		*n*	%	*n*	%	
Alrashed et al. [9]	W 52—mucosal healing	60	59.41	15	51.72	0.46
UST—(*n* = 101)						
VDZ—(*n* = 29)						
Alric et al. [12]	W 14—CRP < 5	25	23.7	39	29.6	0.36
UST—(*n* = 107)	W 48—CRP < 5	31	28.9	29	22	0.25
VDZ—(*n* = 132)						
Bacsur et al. [20]	Biochemical steroid-free remission after induction period	73	45.26	27	41.81	0.66
UST—(*n* = 161)	W 52—biochemical steroid-free remission	65	40.13	23	34.92	0.48
VDZ—(*n* = 65)						
Induction period—16 to 20 weeks						
Biemans et al. [13]	W 12—biochemical SFCR	34	40.5	24	18.9	0.096
UST—(*n* = 85)	W 24—biochemical SFCR	34	40.5	28	21.6	0.21
VDZ—(*n* = 128)	W 52—biochemical SFCR	36	42.1	17	13.2	0.013
	W 12—combined (biochemical and clinical)	4	5.2	7	5.2	1
	W 24—biochemical and clinical	19	21.8	9	7.3	0.77
	W 52—biochemical and clinical	23	27.1	14	10.7	0.031
Yang et al. [7]						
UST—(*n* = 194)	W 26—objective response		54.4		31.1	<0.001
VDZ—(*n* = 84)	W 26—objective remission		11		10	<0.05
Objective remission, endoscopic remission, or normalization of radiography	W 52—objective response		57.8		15.8	<0.001
	W 52—objective remission		27.7		11.2	<0.001
	Endoscopic response at W 26		58.7		40.1	<0.001
	Endoscopic remission at W 26		24.1		12.1	0.001
	Endoscopic response at W 52		60.8		13	<0.001
Endoscopic response; reduction in SES-CD > 50%	Endoscopic remission at W 52		31.5		4.5	<0.001
	Ultrasound response at W 26		62.6		40.7	<0.001
	Ultrasound remission at W 26		19.2		21.5	0.702
	Ultrasound response at W 52		55.8		16.3	<0.001
Endoscopic remission; SES-CD ≤ 2	Ultrasound remission at W 52		29.3		10.9	<0.001
	CT/MR response at W 26		67.5		39.5	<0.001
	CT/MR remission at W 26		17.8		10.1	0.008
	CT/MR response at W 52		61.5		17.9	<0.001
	CT/MR remission at W 52		33.4		7.8	<0.001
Manlay et al. [14]						
UST—(*n* = 88)	W 14—deep remission (SFCR + FC < 100)	16	17.9	3	5.7	0.047
VDZ—(*n* = 45)	W 24—deep remission (SFCR + FC < 100)	23	26.6	7	16.1	0.58

UST: ustekinumab; VDZ: vedolizumab; SFCR: steroid-free clinical remission.

**Table 4:** Findings of studies comparing outcomes of perianal disease.

Study	Findings
Shani et al. [22]	**Patients with active disease:**
	Second-line therapy:
	Ustekinumab: 101/416 patients (24%)
	Anti-TNF: 229/416 (55%)
	Vedolizumab: 55/416 (13%)
	Significantly higher rates of clinical responses (a OR 7.2, 95%CI 3.23–16.06, *p* < 0.001) and remission (a OR 2.2, 95%CI 1.1–4.6, *p* = 0.04)
	**Patients with inactive disease at second-line initiation:**
	Ustekinumab: 17/161(11%)
	Anti-TNF: 83/161 (51%)
	Vedolizumab: 9 (6%)
	No significant difference in recurrence rates of perianal disease (a OR 0.17, 95%CI 0.03–1.09, *p* = 0.06).
Bacsur et al. [20]	Patients with active perianal disease:
	**Ustekinumab**
	*n* = 77/161 (44.7%)
	Symptom improvement at
	W4: 30 (41.67%)
	W52: 39 (54.17%)
	**Vedolizumab**
	*n* = 16/65 (24.6%)
	Symptom Improvement at
	W4: 6 (40%)
	W52 weeks: 5 (33.3%)
	No statistical difference between ustekinumab and vedolizumab
Manley et al. [14]	Patients with active perianal disease
	**Ustekinumab**
	*n* = 39/224 (19.3%)
	Inactive lesions at
	W4: 17/36 (47.2%)
	W54: 14/21 (66.6%)
	**Vedolizumab**
	*n* = 17/88 (19.3%)
	Inactive lesions at
	W4: 9/14 (64.2)
	W54: 3/7 (42.9%)
	No statistical difference between ustekinumab and vedolizumab

**Table 5:** Adverse events among patients treated with ustekinumab and vedolizumab.

Study	Drug	Infection	*p*	Other Adverse Effects	*p*	Total AEs	*p*	AE Requiring Cessation of Treatment	***p***
Alric et al. [12]	Vedolizumab	28.7%		18.9%		47.7%		5.3%	-
	Ustekinumab	11.2%		8.4%		19.6%		0.9%	-
Townsend et al. [8]	Vedolizumab	-		-		-		5.9%	
	Ustekinumab	-		-		-		2.2%	
Biemans et al. [13]	Vedolizumab	-	0.517	-		-	0.464	6%	-
	Ustekinumab	-		-		-		6.9%	
Lenti et al. [15]	Vedolizumab	-	-	-	-	29.6%	-	4.6%	-
	Ustekinumab	-		-		24.5%		2.3%	
Onali et al. [16]	Vedolizumab	3.8%	-	2.7%	-	6.5%		-	-
	Ustekinumab	2%		3.9%		5.9%		-	
Bacsur et al. [20]	Vedolizumab	-	-	-	-	1.5%	-	1.5%	-
	Ustekinumab	-		-		0		0%	
Hyun et al. [10]	Vedolizumab	7.7%	-	9.2%	-	16.9%	-		
	Ustekinumab	12.5%		6.3%		18.8%			
Garcia et al. [18]	Vedolizumab	7.2%	0.9	9.1%	0.4	16.3%		6.2%	
	Ustekinumab	8%		7%		15%		4.4%	
Yang et al. [7]	Vedolizumab	-	-	-	-	6.7%	-		-
	Ustekinumab	-		-		4.9%			
Kappelman et al. [17]	Vedolizumab	5.5%	HR 0.56						
	Ustekinumab	2.95%							

All *p* values > 0.05 with no significant differences in adverse events demonstrated in any of the included studies. - study did not report that specific adverse event.

## Data Availability

The data used in this study was obtained from previously published studies and are available from the original studies.
